# Are decision trees a feasible knowledge representation to guide extraction of critical information from randomized controlled trial reports?

**DOI:** 10.1186/1472-6947-8-48

**Published:** 2008-10-28

**Authors:** Grace Y Chung, Enrico Coiera

**Affiliations:** 1 Centre for Health Informatics, University of New South Wales, Sydney, NSW, 2052, Australia

## Abstract

**Background:**

This paper proposes the use of decision trees as the basis for automatically extracting information from published randomized controlled trial (RCT) reports. An exploratory analysis of RCT abstracts is undertaken to investigate the feasibility of using decision trees as a semantic structure. Quality-of-paper measures are also examined.

**Methods:**

A subset of 455 abstracts (randomly selected from a set of 7620 retrieved from Medline from 1998 – 2006) are examined for the quality of RCT reporting, the identifiability of RCTs from abstracts, and the completeness and complexity of RCT abstracts with respect to key decision tree elements. Abstracts were manually assigned to 6 sub-groups distinguishing whether they were primary RCTs versus other design types. For primary RCT studies, we analyzed and annotated the reporting of intervention comparison, population assignment and outcome values. To measure completeness, the frequencies by which complete intervention, population and outcome information are reported in abstracts were measured. A qualitative examination of the reporting language was conducted.

**Results:**

Decision tree elements are manually identifiable in the majority of primary RCT abstracts. 73.8% of a random subset was primary studies with a single population assigned to two or more interventions. 68% of these primary RCT abstracts were structured. 63% contained pharmaceutical interventions. 84% reported the total number of study subjects. In a subset of 21 abstracts examined, 71% reported numerical outcome values.

**Conclusion:**

The manual identifiability of decision tree elements in the abstract suggests that decision trees could be a suitable construct to guide machine summarisation of RCTs. The presence of decision tree elements could also act as an indicator for RCT report quality in terms of completeness and uniformity.

## Background

### Introduction

Evidence-based medicine (EBM) asks that physicians consult the most current scientific evidence to help them answer clinical questions at the point of care [1,2]. The primary evidence for the efficacy of treatments is often documented in reports of randomized controlled trials (RCTs). While there remains some debate about how applicable RCT results are to the broad population, due to the strict entry criteria required to join them and the confounding effects of co-morbidities with individual patients, good quality RCTs are designed to provide specific and statistically robust answers about the impact of a clinical treatment or intervention on factors such as the patients' prognosis or quality of life. As such, RCTs have a crucial place in the development of the clinical evidence base, and for now are the gold-standard in research design for providing evidence of treatment effectiveness.

While much research has focused on developing technologies to assist clinicians to search for evidence [3-8], there has been little attention paid to the more challenging text-processing tasks of evidence extraction and summarization. As the biomedical literature continues to grow, search technologies will probably need to be augmented with such capabilities, to help identify key points in the documents they retrieve, and ultimately summarize their meaning for busy clinicians.

This paper proposes the use of decision trees as the basis for automatically extracting critical information from published RCT reports. We argue that decision trees, a well-established construct in clinical decision analysis, are an ideal macro-structure for representing and capturing vital elements of RCTs. We report a detailed analysis of the characteristics of a corpus of RCT reports. To assess the feasibility of decision trees as a semantic structure or meaning representation for machine extraction, we examine the quality of RCT reporting, the identifiability of RCTs from abstracts, and the completeness and complexity of RCT abstracts. We perform a series of analyses on a corpus of randomly selected collection of RCT abstracts to answer the following questions:

1. Are RCTs easily identifiable from the abstracts of published reports on Medline?

2. How are RCT abstracts that contain decision tree elements structured? We are interested in the variation in the pre-defined structures and the likelihood that the headings can aid a reader or a computer algorithm in finding decision tree elements.

3. How complex are the decision tree elements in RCT abstracts with respect to study design and language of reporting?

4. How complete is the reporting of decision tree elements within RCT abstracts?

The ultimate goal of our work is to automate the processes involved in creating meta-analyses which are multi-document summaries that bring together the results from multiple RCTs into a single evidence-based recommendation. Our analysis of RCT structure has a further purpose as it suggests a method of automatically testing RCT reports for quality, in terms of completeness, uniformity, and accuracy.

### Information Overload in Evidence-based Medicine

The practice of EBM is hampered by the overwhelming amount of information now available [9]. There are over 200,000 citation entries in PubMed with the Mesh Heading "Clinical Trials". Their publication rate is exponentially rising [10] with over 12,000 trials published in 2007. Furthermore, many have reported that clinicians lack both the time and skills to locate and synthesize the best evidence from the volumes of literature [9,11-14].

One strategy to alleviate this information overload is to create secondary summaries of the evidence such as those produced by the Cochrane Collaboration [15], Evidence-based Medicine [16], the ACP Journal Club [17], and BMJ Clinical Evidence [18]. Evidence-based Medicine and the ACP Journal Club publish reviews that satisfy pre-defined methods criteria. Cochrane and BMJ Clinical Evidence aggregate and distil RCT outcomes into systematic reviews and meta-analyses to guide best practice [19]. In particular, *statistical meta-analysis *is one of the most powerful tools for deriving meaningful conclusions from sometimes conflicting reports and generating statistical power greater than that of the individual studies [20-22].

Creating a systematic review requires a team of experts to exhaustively sift through trial databases and published reports of RCTs. The Cochrane Collaboration, arguably the largest and best-equipped international organization focused on extracting best clinical practice from research practice, requires systematic reviews to meet stringent criteria when selecting studies and collecting and analyzing results. By 2000, the Cochrane collaboration had produced 795 systematic reviews covering 12,000 clinical trials. Yet there had been 200,000 new trials added to its database [23]. Clearly, the volume of published research is growing at a rate that exceeds the human resources available to systematically and comprehensively synthesize it [24].

This problem is further compounded as numerous studies have identified deficiencies in the completeness and accuracy of some RCT reports [25-28] and as a result, there have been concerted efforts to standardize the reporting of clinical trials. Trial Bank [29] encourages registration in a database through manual entry of descriptions of design and execution, but only a small number of trials have been archived so far. The CONSORT statement [30,31] is gaining currency as an effort to improve the reporting of clinical trials through a checklist of 22 items and a participant flow diagram.

Automated text mining and natural language processing now holds the promise for easing the burden of effectively summarizing such large volumes of medical knowledge. An ongoing stream of research into summarization of biomedical research papers is tied to the resource intensive manual mark-up and extraction methods. Georg et al. [32] extend the well known Guideline Elements Model (GEM) tool kit for medical document mark-up to support the manual mark-up and extraction of *if-then *rules from clinical guidelines. Aguirre-Junco et al. [33] take a similar approach using mark-up tools to extract decision trees, again from guidelines which already summarize expert consensus.

Text mining for EBM is a growing area of research, with researchers developing semantic search and evidence summarization techniques for reranking search results and locating and synthesizing clinical answers for clinical practice [4,34-36]. The focus of all these efforts is on supporting clinicians, and to our knowledge, no technology support has been proposed that can assist systematic reviewers to produce summaries and meta-analyses. Demner-Fushman et al. [35] have used the PICO (Population/Problem, Intervention, Comparison, Outcome) Framework as a basis for extracting clinically relevant information from Medline abstracts from core clinical journals. PICO [37] was originally conceived as a method to reformulate clinical problems into "well-built" questions that can be passed on to an information retrieval engine, leading to improved precision and recall for finding clinical answers. However, it has been reported that questions posed by clinicians are not generally amenable to a PICO formulation, and moreover, rephrasing questions does not increase success rates in finding answers [38,39]. In recent work, Dawes et al. [40] investigated the identifiability of PECODR (Patient-Population-Problem, Exposure-Intervention, Comparison, Outcome, Results) elements, an extension of the PICO framework, in medical journal abstracts.

In text mining, the granularity at which information should be extracted is often a subject of debate. It is clear that a complete semantic interpretation of language is beyond the reach of current methods. In the clinical domain, researchers have predominantly utilized machine learning algorithms, particularly statistical classifiers to extract information at the sentence level usually within the abstract [35,41-43]. In some cases, researchers pinpoint factual information within a document by identifying textual passages that follow scientific arguments such as Purpose, Interpretation and Findings [44].

In our work, we attempt to exploit the strict design principles and stringent reporting guidelines for RCTs [45] which provide us with domain-specific elements that can act as semantic constraints for automated text extraction algorithms. Further, successful machine extraction of the RCT elements from a text could be used to signify a well-written report or well-conducted trial, and could thus be used as an automated quality assurance mechanism for assessing the quality of RCT reports.

### Decision Trees: Macro-structures representing RCTs

Medical decision science has long utilized decision trees as the main representation for modelling decision choices, and there is a substantial body of research behind these trees making them general and powerful constructs [46].

A decision tree [47] is a mathematical and visual representation of all possible decision options for a given choice, and the consequences that follow each, usually expressed in terms of likelihoods and utilities. For an RCT, a decision tree could compare the outcomes of competing therapies for a given clinical condition.

**Figure 1:**
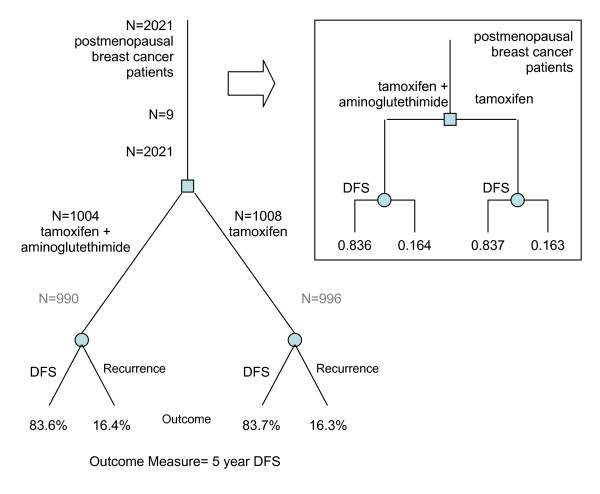
**Example tree structure from an RCT (PMID: 12637461)**. The tree compares tamoxifen treatment with tamoxifen plus aminoglutenthimide for postmenopausal breast cancer patients. The primary outcome measure is 5 year disease free survival (DFS), whose numerical values can directly be utilized in decision analysis. The corresponding true decision tree is illustrated.

Conventionally, decision trees are constructed by analyzing the primary literature, which provides estimates for event likelihoods. Where patient utilities are required, standard methods are available to elicit robust numerical estimates [47]. The experimental design of RCTs makes them particularly amenable to be recast in decision tree form. In Figure 1, we illustrate the derivation of a decision tree from the participant flow diagram of an RCT, which provides the tree structure, and the RCT outcomes, which provide values for the outcome nodes of the tree. The participant flow tree is determined by the intervention and comparison experimental design, and the outcomes measured for each patient group. It includes details such as total participant numbers enrolled, excluded and assigned as well as the number of evaluable patients. For each comparison group, chance nodes split the tree to report different outcome events. The derived decision tree excludes participant information, and converts outcome measures into a likelihood or probability.

**Figure 2:**
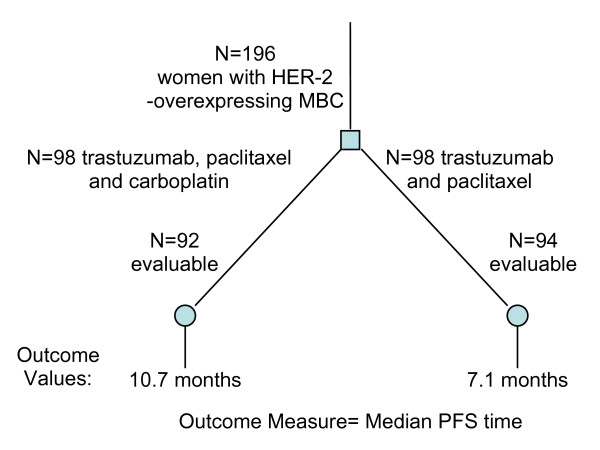
**Example intermediate decision tree derived from an RCT (PMID: 16782917)**. The primary outcome measure is the median Progression Free Survival (PFS) time, computed for each group in each treatment arm. The output decision tree taken directly from the article is an intermediate representation as opposed to a true "decision-ready" tree structure.

The decision tree structure will vary according to the number of randomized treatment groups or arms. A new tree can be drawn for each primary or secondary outcome that is analyzed. Whether or not a true decision tree can be derived from the RCT report depends on how the outcomes are defined. A common way of measuring outcome is a binary measure (whether or not an event has occurred). As in Figure 1, the resultant values compared are frequency counts of occurrences at each arm which could be converted to predictive probabilities, and thus producing a "true" decision tree. Many other ways of measuring outcomes yield numerical values for each treated subject, for instance, time to an event, continuous values from physiological measurements, discrete numerical values from frequency counts of events or standardized scales (e.g. quality of life ratings). The tree structures derived for these outcomes will be intermediate representations from which further statistical analyses are required to convert outcome values to probabilities to populate a true "decision-ready" tree. Figure 2 illustrates one example of such an intermediate decision tree.

The decision trees described above can have a dual use. For systematic reviewers, decision trees can answer questions about study design and detail intervention outcomes. Extracted decision trees from multiple studies may even be combined to help expert reviewers conduct meta-analyses by synthesizing outcomes. For medical practitioners, decision trees can be directly integrated into decision support systems used at the point of care.

## Methods

### Corpus Collection

A corpus of RCT abstracts was compiled by conducting a PubMed search, specifying RCT in the publication type field. To obtain a representative cross-section of clinical conditions, the following keywords were used: asthma, diabetes, breast cancer, prostate cancer, erectile dysfunction, heart failure, cardiovascular, angina. This yielded 7620 abstracts (Group A).

A smaller subset of 455 abstracts (Group B) was randomly selected for detailed analysis and annotation. These abstracts were sourced from 197 different journals dating from 1998 and 2006.

### Identifiability of RCTs: Categorization of Studies Labelled as RCTs

Not all the documents labeled as RCTs by Medline are genuine RCTs. To determine whether RCTs can easily be found via PubMed, we manually labeled the RCTs in Group B. Abstracts were first divided into Group R (primary RCT reports, and excluding studies that were not truly randomised e.g. quasi-random methods of allocation) and Group N (publications that are not primary reports), and further subdivided into the following six categories:

• R_1_: Primary reports of single RCTs with full descriptions of study design and outcomes.

• R_2_: Primary reports of RCTs with a complex sequence of intervention or randomization phases or a complex combination of multiple RCTs.

• R_3_: Sub-studies of RCTs, which generally include descriptions of study design, and focus on specific outcome measures and data analyses. Reports in this group (follow-up reports, updates, sub-group and post-hoc analyses) often describe larger scale studies whose methodologies (recruitment etc) have been described in detail in previous reports.

• N_1_: RCT protocols or announcements, often with only partial description of recruitment strategies, baseline characteristics and methodology with no results.

• N_2_: Systematic reviews of one or more trials, including pooled analyses.

• N_3_: Other studies, (population studies, retrospective reviews and non-randomized studies).

The manual categorization was performed by GYC. A random subset of 50 articles was assigned to a domain expert (a systematic reviewer) to arrive at category definitions and perform an inter-rater reliability assessment, measured by Cohen's kappa [48]. During categorization, all abstracts were assumed to be primary reports of RCTs unless it was apparent in the abstract that they were not.

### Analysis of Structured Abstracts

The variation in the structured abstracts in both the large corpus (Group A) and the hand labeled primary RCT abstracts (R_1_) were examined. Abstracts were first classified as structured or unstructured, based on the presence of labeled headings appearing within the structured group. Abstracts identified as being structured were further analyzed to identify the number and type of unique headings used, and variations in the order of reporting of these subheadings. Some headings differed in the terms they contained, but were otherwise semantically identical (e.g. "Setting", "Study Setting"). To assist with analysis, the full list of unique headings was condensed into a shorter list of semantic equivalent heading classes.

### Study of Decision Tree Elements: Complexity, Completeness and Identifiability

Abstracts in R_1 _were analyzed, looking for the presence, location, and type of key decision tree elements. Conventionally, RCTs can be distinguished by many elements that follow the well-documented principles of sound trial design [45]. Examples are the design of the randomized assignment (conventional, crossover, parallel group, 2 × 2 factorial) or the method of blinding (single or double blinded, open label). These study design variations are generally recognizable from the key words in the descriptions. The adequacy in their reporting have been explored elsewhere [25,26]. From the standpoint of automatic processing, we are presently interested in characterizing how the comparison of interventions, the assignment of population groups and measurement of outcome values are reported. For each of the three elements, we examine the variation in complexity across our set of RCTs, the variation in completeness of their reporting in abstracts and if they are identifiable particularly in structured abstracts. Along each of the above dimension, each abstract is assigned to several subcategories.

For intervention information, abstracts in R_1 _were assigned to two subcategories:

I_1_: Pharmaceutical interventions where drugs are compared in each arm. A placebo is often allocated at one arm.

I_2_: Non-pharmaceutical interventions such as surgical procedures, behavioral therapies, or multi-modal strategies. A control or usual-care group is often allocated at one arm.

For population information, the complexity of population assignment in the study design was studied. Each abstract in R_1 _was assigned to the following:

P_1_: A single patient group defined by some criteria, with subjects randomly assigned to each treatment arm.

P_2_: A single patient group with one (or more) additional non-randomized control group(s), corresponding to additional arm(s) in the tree. For instance, one control group can be age and gender matched healthy cohorts.

P_3_: Multiple patient groups defined according to some criteria prior to randomization. Results for each patient group are usually analyzed together and separately, and compared.

For outcome information, the following categories were given to the abstracts:

O_1_: One or more explicit primary outcome variables are identified from the abstract, including the outcome values to form decision trees.

O_2_: Partial values of the primary outcomes are identified from the abstract and the remainder can be elicited from the text of the article to complete decision trees.

O_3_: One or more explicit primary outcome variables are identified from the abstract, but no numerical values are provided and only qualitative statements about polarity of outcomes.

GYC performed category assignments for abstracts in R_1_. A team of three clinically trained annotators identified the interventions being compared, and the population counts (the total number of subjects, the number assigned at an arm of the study and the number lost to follow up or drop outs) from the texts. The sentences which describe the assignment of each treatment to each comparison group are also labeled, as these sentences would be the basis for any extraction of information to create a decision tree. Annotators underwent training sessions using test abstracts to ensure common understanding of sentence classes. Where there was a disagreement about sentence annotation class within a given abstract, a consensus annotation was agreed. For outcomes, a randomly selected subset of 21 abstracts is analyzed and the outcome values are labeled. For each of the three elements (intervention, population group and outcome), a qualitative analysis was performed, examining the distribution of information across headings used within the structured abstracts.

## Results

### Identifiability of RCTs: Categorization of Studies Labeled as RCTs

The number of abstracts in each subcategory of 455 Group B abstracts is summarized in Table 1. Within this corpus, 86% (391/455; Group R) were identified as RCTs and 14% (64/455; Group N) were not strictly primary RCT reports. Most of the RCT reports were primary reports of single RCTs (73.8%; Group R_1_). The studies in Group N were predominantly population studies within subgroup N_3_. Inter-rater agreement (Cohen's kappa [48]) for this assignment task was 0.826 (25 in disputed set), and a final agreement was arrived at with the disputed set.

**Table 1:** Number of abstracts in each subcategory in a randomly selected corpus of 455 RCT abstracts.

**Classes**	**Total**	**R_1_**	**R_2_**	**R_3_**	**N_1_**	**N_2_**	**N_3_**
		**Single RCT**	**Complex RCT**	**RCT substudy**	**RCT protocol or announcement**	**Systematic review**	**Other study**

**N (%)**	455	336 (73.8%)	21 (4.6%)	34 (7.5%)	5 (1.1%)	6 (1.3%)	53 (11.6%)

Only a small portion (4.6%; R_2_) of the RCTs were complex reports of multiple RCTs. Some of these contained multiple randomization phases within a single study, together with multiple patient groups. It was often difficult to ascertain from these abstracts the number of treatment arms or precisely which stages of the study were randomized and which were not, and reporting of outcomes are equally complex e.g.:

*"At baseline, 294 subjects were randomized to receive either placebo first (n = 139) or inactivated trivalent split-virus influenza vaccine first (n = 155). Study subjects were categorized into 2 groups: subjects in group 1 (n = 148) were receiving medium-dose or high-dose inhaled corticosteroids (ICSs) or oral corticosteroids, whereas subjects in group 2 (n = 146) were not receiving corticosteroids or were receiving low-dose ICSs. ... Serologic responses to each influenza vaccine antigen were significantly higher in vaccine than in placebo recipients and were similar among influenza vaccine recipients in groups 1 and 2 for the following endpoints: rise in antibody titer, percent of participants who developed a serological response, and percent of subjects who developed a serum hemagglutination inhibition antibody titer > or =1:32." PMD: 15100679*.

The descriptions in R_3 _(RCT substudies) varied considerably because this group encompasses a large variety of studies. These include updates of results, follow-on studies, or post-hoc analyses which could include comparisons of subgroups or analyses of secondary outcomes.

### Analysis of Structured Abstracts

Over half the abstracts were structured, in Group A (58%; 4441/7620) and in the primary RCT reports Group R_1 _(68%; 230/336) (Table 2). In Group A, there were 238 unique section headings which were however often semantically equivalent, and could be manually mapped to each other. Manual mapping into equivalence classes condensed the section headings into 106 classes. Examples of these mappings are shown in Table 3.

**Table 2:** The number of unstructured and structured abstracts in Group A, the original set of abstracts and Group R_1_, the primary RCT reports from the randomly selected subset.

	**Number of Abstracts in A**	**Number of Abstracts in R_1_**
Total abstracts	7620 (100%)	336 (100%)

Unstructured abstracts	3179 (42%)	106 (32%)

Structured abstracts	4441 (58%)	230 (68%)

Structured abstracts with explicit heading for intervention	283 (3.7%)	14 (4.2%)

Structured abstracts with explicit heading for population	433 (5.7%)	17 (5.0%)

Structured abstracts with explicit heading for outcome measures	329 (4.3%)	18 (5.3%)

Structured abstracts with explicit heading for all three subheadings	162 (2.2%)	11 (3.3%)

**Table 3:** Examples of equivalence classes of pre-defined sub-headings in structured abstracts.

**Class**	**Example Heading Names**
Aim	Goal, Aim of the study, Purpose

Setting	Setting, Study setting, Settings and Location

Participants	Study population, Type of participants, Patients or participants, Sample

Outcome measure	Measurements, Primary outcome measure, Study endpoints, Major outcome measures

After class mappings, there were 400 different sequence patterns in the combinations of section headings, with over 90% of these variations occurring less than 10 times. The most common section heading patterns, and some rarer patterns, are shown in Table 4. The variation in structural ordering was large, and many of the heading names were unique. A typical heading sequence is "Background, Aim, Methods, Results, Conclusions" but there were many compound headings such as "Method/Results", "Results/Conclusion", "Subjects/Settings" etc. Most of the variation in heading substructure was generated in reporting the breakdown of experiment design, for example, "Subjects", "Setting", "Patients", "Intervention", "Outcome Measures" etc. The exact sequence and combination of these headings differ greatly across abstracts. In Table 2, the number of abstracts containing a distinct heading for intervention, population and outcome measures are reported for Group A and R_1_. Only a very small portion of abstracts have all three distinct sub-headings (2.2% of Group A and 3.3% of R_1_).

**Table 4:** Examples of the patterns that occur in the section headings of structured RCT abstracts of Group A.

**Structure of Abstracts**	**% of Corpus**
Background, Method, Result Conclusion	16

Aim, Method, Result, Conclusion	14

Aim, Patient and Method, Result, Conclusion	8.5

Background, Aim, Method, Result, Conclusion	7.6

Background, Method and Results, Conclusion	6.6

Aim, Participants, Design, Measurements, Result Conclusion	< 1

Context, Design, Setting, Participants, Outcome Measures, Result, Conclusion	< 1

Aim, Design and Setting, Participants, Intervention, Measurements and Main Results, Conclusion	< 1

### Study of Decision Tree Elements: Intervention Information

Of the abstracts in R_1 _(single RCT report), 63% of interventions were pharmaceutical (I_1_) and 37% non-pharmaceutical (I_2_) (Table 5). In terms of complexity, most abstracts reported only 2 treatment arms in the study. (76% in I_1 _and 78% in I_2_). In terms of completeness, only one abstract in each subgroup gave no indicator of the number of treatment arms in the study.

**Table 5:** Intervention information for primary RCT abstracts (R_1_).

	**Pharmaceutical intervention (I_1_)**	**Non-pharmaceutical intervention (I_2_)**
Total abstracts	213	123

Number of treatment arms unknown	1	1

2 treatment arms	161	96

3 treatment arms	33	20

4 or more treatment arms	5	6

In many abstracts, multiple sentences were identifiable as intervention sentences. Of the 106 structured abstracts in R_1_, there are 254 intervention sentences. Table 6 reports the location of the sentence(s) describing assignment of intervention at each arm within the structured abstracts in R_1_, with respect to the mapped heading class.

**Table 6:** Distribution of sentences describing interventions and comparisons with respect to the classes of pre-defined subheadings in primary RCT structured abstracts (R_1_).

**Heading Class**	**Number of Intervention Sentences**
Method	197 (78%)

Intervention	19 (7.5%)

Aim	14 (5.5%)

Method and Results	12 (4.7%)

Design	8 (3.1%)

Results	2 (0.1%)

Background	1 (<0.1%)

Setting	1(<0.1%)

The intervention was described in the Method section (78%), in Aim (5.5%), Design (3.1%) or Results (0.1%). When there is no method section, 30% of intervention sentences appear in the Aim and 60% in Design. The intervention is mentioned 100% of the time under Intervention if that heading exists. Even when there is a Method section, the explicit mention of intervention is not guaranteed to be there.

We perform a qualitative analysis of the language used to describe the comparison of intervention. Most (96.7%; 206/213) abstracts in I_1_(pharmaceutical intervention) describe the randomized assignment of treatments within a single sentence, e.g.:

*"Following a 3-week, single-blind placebo run-in period, eligible patients were randomized to receive either manidipine 10 mg or enalapril 10 mg once daily for 24 weeks." PMID: 15811479*.

These intervention sentences tend to embed additional information about methodology such as duration of run-in period or treatment period and dosage. They occur frequently as compound noun phrases. The actual comparison details can be described in parenthetical remarks e.g.:

*"Patients were randomly allocated to treatment with talinolol (100, 200 or 300 mg once daily) or placebo .." PMID:15726874*.

The description of assignment of treatments to various groups was often more complex in I_2 _(non-pharmaceutical intervention), using multiple sentences. There were 18 abstracts (14.6%; 18/123) which describe randomization and assignment to groups in 2 to 5 sentences e.g.:

*"Diet intervention was performed by telephone counseling and promoted a low fat diet that also was high in fiber, vegetables and fruit. The comparison group was provided with general dietary guidelines to reduce disease risk .." PMID: 11148556*.

Of the remaining I_2 _abstracts (85%; 105/123), assignment of intervention is described in one sentence. Among these, the specification of the intervention procedure varied in detail and was often underspecified. The actual intervention procedure might be described further in the abstract or elsewhere, possibly only in the article because these interventions can be quite complex to describe in full in the abstract e.g.:

*"Forty-four women were randomly assigned to receive either a lifestyle change or a lifestyle change with self-control skills intervention." PMID: 15186658*.

A substantial number of I_2 _abstracts (19.5%; 24/123) mention the assignment of intervention in a sentence, followed by one or more sentences which describe the groups, e.g.:

*".. 277 women diagnosed with CVD (mean age 61 +- 10 years) were randomly assigned within 1 of 12 San Francisco Bay Area hospitals to a usual-care group (UG; n = 135) or intervention group (IG; n = 142). ..The UG received strong physician's advice, a self-help pamphlet, ... The IG received strong physician's advice and a nurse-managed cognitive behavioral relapse-prevention intervention at bedside ..." PMID: 14769679*.

### Study of Decision Tree Elements: Population Information

The population subcategories reflect on the complexity of RCT study designs and reporting. Table 7 reports the numbers abstracts within each subgroup of population characteristics. 95% (320/336 in P_1_) of these are simple RCTs with a single population group with subjects randomly assigned to two or more treatments.

**Table 7:** Number of abstracts in each sub-category for population treatment for 336 primary RCT abstracts (R_1_)

	**single patient group randomized (P_1_)**	**single patient group randomized, additional control group (P_2_)**	**more than one patient group randomized (P_3_)**
Number of abstracts	320	11	5

Reporting of patient characteristics in P_2 _and P_3 _was more complex because abstracts included the number of patients, age and gender, inclusion criteria and sometimes baseline characteristics are for each group. The outcomes for the groups might be analyzed separately or together. Effectively this would result in a distinct decision tree for to each population group e.g.:

*"A total of 244 T3 – 4 M0 patients and 200 T1 – 4 M1 patients were randomized to either OE or PEP therapy. .. In the T3 – 4 M0 patients, the treatment (PEP versus OE) and the presence of pretreatment vascular diseases were statistically significantly associated with a risk of CV complications (p = 0.01 and 0.003, respectively). In the T1 – 4 M1 patients, such an association was not found." PMID:1626166*.

The completeness in the report of population counts was also studied. Most abstracts (84%) specified the total number of participants in a study (Table 8). However, whether the number refers to the population at recruitment, enrolment, assignment or completion varied from one abstract to another. It can be expected that the numbers at each stage of the trial are different, but this information is often ambiguous in the abstract or buried within the text of the article. At times the stage of the trial (enrolment or evaluation etc) associated with the population number is not specified. In the following example, population information was given under the Patients subheading:

**Table 8:** Overall population information for primary RCT abstracts (R_1_).

	**Number of abstracts**
Abstracts reporting total subjects in study	280 (84%)

Abstracts reporting subjects assigned to each arm	122 (36%)

Abstracts reporting the number of drop outs	5 (1.5%)

No information about population	20 (6%)

*"Patients: Twenty-five mono-opioid addicted patients with mild to moderate systemic disease (ASA II classification) in a methadone substitution program." PMID:10809268*.

Information that specifies the number of evaluable patients at follow-up is critically important in determining outcomes. Of the 280 abstracts that provide the total number of subjects, only 50 of them explicitly report the number of "evaluable" patients at completion or follow-up. Of the 50, 40 report two or more numbers, referring to assignment or enrolment as well as follow-up, and 10 report only the number at completion.

From Table 8, 122 (36%) abstracts report the number of patients allocated to each arm of the trial. Of the 214 abstracts that do not report numbers at each arm, 46 have crossover designs.

Table 9 shows the location of the annotated instances of population values in structured abstracts, revealing that population information is most often found in the Method section or sections related to experimental design, although it is not universally the case.

**Table 9:** Distribution population information with respect to the classes of pre-defined subheadings in structured abstracts.

	**Total number of instances of population values**
Method	195

Intervention	3

Aim	3

Method and Results	22

Design	16

Results	82

Patients	15

### Study of Decision Tree Elements: Primary Outcomes Measures and Values

Of the 21 abstracts examined, 15 abstracts (71%) indicate the primary outcome measures as well as the values so that a decision tree could be elicited directly (Table 10). In 7 of the 15 abstracts in O_2 _(partial outcome measures and values), the primary outcome measures were binary events, and could be converted to probabilities for decision analyses. Example outcome events included: objective response rate, progression-free survival, 5-year disease free survival, clinical benefit rate. For 8 of the 15 abstracts the primary outcome measures could not be directly convertible to probabilities. Example outcome measures included: treadmill exercise time, measurements with biomarkers such as blood pressure, glucose levels. In general, outcomes can be reported as the differential in the measurement, given as a percentage or a literal value, along with a (usually 95%) confidence interval and/or a p value. Otherwise the measurement (as a mean and standard deviation) at baseline and at follow-up can be provided for each group. Often, additional statistical measures are given such as odds or hazard ratio. In all, the outcomes can be reported in multiple sentences or in a single sentence with numerical values within parenthetical remarks. e.g.:

**Table 10:** Number of abstracts in each sub-category for reporting of outcomes

	**O_1_** Full primary outcome measures and values	**O_2_** Partial primary outcome measures and values	**O_3_** numerical outcomes not translatable into a chance node
Number of abstracts	15	2	4

*"Baseline values were: 24 hour UAE [geometric mean (95% CI)] 134 (103 to 170) mg/24 hours, ambulatory blood pressure [mean (SD)] 140 (10)/77 (7) mm Hg, and GFR 103 (19) mL/min/1.73 m2. Reductions in UAE from baseline were 52% (46% to 57%), 49% (43% to 54%), and 59% (54% to 63%) with increasing doses of irbesartan (P < 0.01)." PMID: 16105050*.

4 abstracts provide only qualitative descriptions of the effect of an intervention, sometimes with p values e.g.:

*"Main Outcome Measures: Any limitation in activity was the primary outcome.. Results: After adjusting for covariates, the odds of having any limitation in activity during the 90 day trial were significantly (P = .03) lower for children randomized to the Health Buddy." PMID: 11814370*.

In all cases, outcome statements with numerical values are found in the Results sections of structured abstracts.

## Discussion

Our initial analysis has shown that decision trees elements are manually identifiable from RCT abstracts; that for the majority of them, the study design can in principle be extracted as a decision tree, and that some complete decision trees are indeed extractable from RCT abstracts.

### Identifiability of Abstracts

Most abstracts found in the documents we retrieved from the Medline database were primary reports of RCTs, but it is clear that a simple search of RCT reports yields results that are corrupted by other types of studies. To increase precision in the search for RCTs, more complex search filters are needed to exclude trials that are not genuine RCTs.

There is mixed prior evidence that structured abstracts [49] improve information retrieval and readability as intended [50-52]. Some have reported that abstract structure can be inconsistent with missing sections [53-55]. We have found here that the sequence of pre-defined headings varies widely, and the location of the critical elements for RCTs such as the comparison of intervention and population numbers cannot be reliably located according to the names of sub-headings. Specifically relevant headings such as Intervention, Patients, Outcome Measures, are not rare. In the past, researchers have used machine learning methods to classify sentences in unstructured abstracts according to the generic headings of Aim, Method, Results and Conclusion [41-43]. We speculate that like structured abstracts, key facts in unstructured abstracts such as intervention and population are not necessarily located in what would be considered a Method sentence.

### Complexity

A sizeable portion of our corpus of studies consists of simple RCTs with a single population group assigned to two or more interventions. The reporting of pharmaceutical interventions appears to be simpler and more consistent than non-pharmaceutical interventions in the abstract. The reporting of outcomes can also vary in complexity, depending on the amount of detail provided in the abstract. An automatic extraction algorithm would need to interpret numerical values and assign the correct set of measurements to each respective arm.

For RCTs that concern more than a single population (P_2_) or a complex sequence of treatments or randomization phases (R_2_), the decision tree could be multi-layered with separate branches for each population group. This would pose a more challenging problem from an automated extraction point of view because of the many different configurations that are possible.

### Completeness

For primary RCT abstracts (R_1_), a simple decision tree representation could at least be partially instantiated. Information about the comparison of intervention can be found in the abstract but there is more variation in reporting of population counts in terms of completeness. In our examination of outcome values, most abstracts provide the numerical values for the primary endpoints although a few only provide qualitative or interpretative statements of their findings.

From the standpoint of automated processing, it will at least be necessary to use the full text for complete decision trees in many cases, particularly if all decision trees with respect to all endpoints or assessments are desired. However, it is beyond our scope to assess the difficulty of extracting complete decision trees from the full text. It will also be necessary for an automated algorithm to disambiguate among all the population counts that are given in order to interpret a trial properly and to infer which critical pieces are in fact missing from the reporting.

### Quality of Reporting

There is mixed evidence of the impact of efforts such as CONSORT on the quality of RCT reporting [29,32,56,57]. It may be possible that further to CONSORT, another metric for measuring the quality of reporting is whether full decision trees can be elicited from the abstract (by hand or by machine), particularly for primary reports of simple RCTs. We argue that inclusion of elements of decision trees could be a reasonable additional prescription for what should be reported in RCT abstracts. For instance, the explicit inclusion of factors, such as population counts at each stage and numerical outcome measurements corresponding at each arm, would not only improve readability but would ease the task of automatic information extraction that could lead to applications that enhance semantic search and automatic summarization.

### Limitations

In proposing the use of decision trees as an underlying semantic representation, this study has outlined a basic approach to representing the critical elements of interest in RCT studies. However, reviewers may need to examine many other methodological details such duration of trial, follow up period, secondary outcomes such as adverse events, toxicity, and side effects, or statistical computations such as odds ratios, hazard ratios, and so on. These are factors which should ideally be automatically extracted in a text processing system as well as the basic decision tree structure in order to answer all the questions that may arise.

In our analysis, the authors automatically computed the number of structured abstracts in Group A by a method that uses regular expressions to look for section headings. This method was not evaluated and some errors may potentially exist.

Our data analysis is a preliminary study to characterize RCT reports across a set of typical conditions. A more detailed analysis using larger data sets may provide more insight into the reporting of factors such as intervention, outcomes and population in abstracts. An extensive study of full articles is also necessary to reveal whether this information can be extracted reliably. A larger data set would be necessary for full annotation for the purpose of training classifiers for machine extraction. Finally, many RCTs are not indexed in the Medline database and we recognize that our data set may not be representative of all the data available to systematic reviewers.

## Conclusion

This paper has proposed the use of decision trees as representation of RCT reports to support automated extraction of the critical elements of RCTs, and subsequent machine summarization.

In an analysis of a corpus of randomly selected abstracts, we found that decision tree elements can be elicited manually from the majority of RCTs returned from a search on Medline. We have also suggested that a complete report of these parameters in RCT abstracts is an important quality measure for comprehensibility for humans and processing by machine. We are currently in the process of developing annotation guidelines and annotating components of decision trees from the full text of RCT reports. Future work will be the implementation of a system for automatically extracting decision tree components and presenting the results in a graphical format.

## Competing interests

The authors declare that they have no competing interests.

## Authors' contributions

Author GYC designed and conducted the data collection, annotation and analysis. EC conceived the use of decision trees as a knowledge representation for randomized controlled trial reports. GYC wrote the manuscript with extensive feedback from EC.

## Pre-publication history

The pre-publication history for this paper can be accessed here:


